# Machine Learning Isotropic *g* Values of Radical Polymers

**DOI:** 10.1021/acs.jctc.3c01252

**Published:** 2024-03-08

**Authors:** Davis Thomas Daniel, Souvik Mitra, Rüdiger-A. Eichel, Diddo Diddens, Josef Granwehr

**Affiliations:** † Institute of Energy and Climate Research (IEK-9), Forschungszentrum Jülich GmbH, 52425 Jülich, Germany; ‡ Institute of Technical and Macromolecular Chemistry, RWTH Aachen University, 52056 Aachen, Germany; § Institute of Physical Chemistry, University of Münster, 48149 Münster, Germany; ∥ Institute of Physical Chemistry, RWTH Aachen University, Aachen 52056, Germany; ⊥ Helmholtz Institute Münster (IEK-12), Forschungszentrum Jülich GmbH, 48149 Münster, Germany

## Abstract

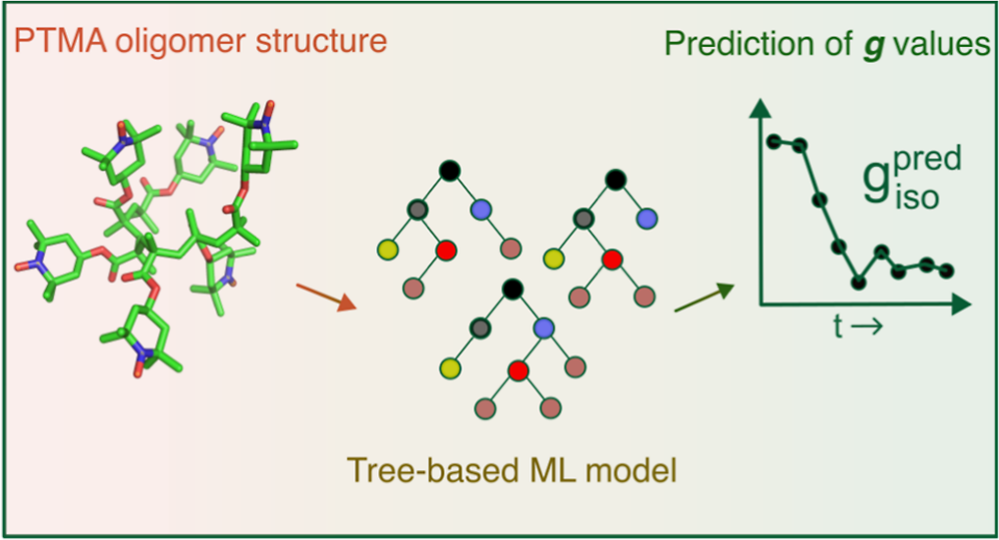

Methods for electronic structure computations, such as density functional theory (DFT), are routinely used for the calculation of spectroscopic parameters to establish and validate structure–parameter correlations. DFT calculations, however, are computationally expensive for large systems such as polymers. This work explores the machine learning (ML) of isotropic *g* values, *g*_iso_, obtained from electron paramagnetic resonance (EPR) experiments of an organic radical polymer. An ML model based on regression trees is trained on DFT-calculated *g* values of poly(2,2,6,6-tetramethylpiperidinyloxy-4-yl methacrylate) (PTMA) polymer structures extracted from different time frames of a molecular dynamics trajectory. The DFT-derived *g* values, *g*_iso_^calc^, for different radical densities of PTMA, are compared against experimentally derived *g* values obtained from *in operando* EPR measurements of a PTMA-based organic radical battery. The ML-predicted *g*_iso_ values, *g*_iso_^pred^, were compared with *g*_iso_^calc^ to evaluate the performance of the model. Mean deviations of *g*_iso_^pred^ from *g*_iso_^calc^ were found to be on the order of 0.0001. Furthermore, a performance evaluation on test structures from a separate MD trajectory indicated that the model is sensitive to the radical density and efficiently learns to predict *g*_iso_ values even for radical densities that were not part of the training data set. Since our trained model can reproduce the changes in *g*_iso_ along the MD trajectory and is sensitive to the extent of equilibration of the polymer structure, it is a promising alternative to computationally more expensive DFT methods, particularly for large systems that cannot be easily represented by a smaller model system.

## Introduction

Electron paramagnetic resonance (EPR) spectroscopy, a technique to characterize systems with unpaired electrons, is highly effective in structure elucidation and mechanistic investigations when coupled with theoretical modeling.^1,2^ While EPR selectively probes unpaired electrons and can be used to gain insights into the electronic structure of spin systems, electronic structure methods such as density functional theory (DFT) enable the calculation of EPR spectroscopic observables and confirm experimental inferences made by using EPR methods. In the case of small molecules, DFT techniques are a popular choice for accurate calculations of physical and chemical properties at moderate computational costs.^3,4^

A combination of DFT and molecular dynamics (MD) is frequently employed for simulating EPR spectra of dynamic systems, and EPR parameters calculated using DFT methods often serve as initial values for least-squares fitting of EPR spectra.^5^ In order to fully capture the evolution of a specific property along an MD trajectory, a large number of DFT calculations are required. For larger systems, DFT calculations can become considerably more expensive, requiring high-performance computing systems to afford such calculations. As a viable alternative, machine learning (ML) methods have seen a recent surge in application, especially in materials^6^ and chemical sciences.^7,8^ ML algorithms can be used to efficiently learn structure–property correlations and make predictions for unknown structures with comparable accuracy as DFT at a fraction of the computational time. Various physical and chemical properties including chemical shifts,^9^ ground-state energies,^10^ or redox potentials^11^ have been targeted using ML methods.

A key parameter derived from EPR spectra is the *g* value, which describes the interaction of the electron spin with the applied magnetic field. The observed *g* value deviates from the free electron *g* value (*g*_e_ = 2.00232) mainly due to spin–orbit coupling, which is a relativistic effect arising from the interaction of spin and orbital angular momentum. Spin–orbit coupling is more pronounced for heavier elements. Transition metal ions can show large deviations from *g*_e_, while organic radicals show smaller deviations in comparison.^12^ Therefore, the *g*-shift is characteristic of the chemical identity of the system. In the solid state and frozen solutions, the value of *g* is dependent on the orientation of the molecule with respect to the externally applied magnetic field, leading to *g* anisotropy described by a **g** tensor with three principal values: *g*_1_, *g*_2_, and *g*_3_.^13,14^ In the liquid state, the anisotropy is averaged out due to fast tumbling of molecules, and an isotropic *g* value, *g*_iso_ = (*g*_1_ + *g*_2_ + *g*_3_)/3, is obtained. As **g** is related to the underlying molecular geometry, *g* values can be used to investigate electronic distributions in molecules. This is particularly relevant in the case of transition metal complexes, where an analysis of the **g**-tensor components can be used to study geometrical distortions.^15^ For organic radicals, *g* values can be a sensitive probe for the identification of the radical center and elucidation of structural changes in its environment.^16−18^ The magnitude of *g* value shifts as a result of environmental changes in organic radicals can be minimal, usually of the order of 10^–4^, and high-field EPR measurements are used to gain the required spectral resolution.^17,19^

In systems with high spin concentration and small interspin distances, spin–spin interactions such as the Heisenberg spin exchange or dipole–dipole interactions considerably affect the EPR spectrum. As a consequence of strong exchange, the coalescence of spectral features may occur and the EPR spectrum is mainly characterized by *g*_iso_ and the line width.^20^ Prominent examples of such systems include organic radical polymers (ORPs), where a high radical concentration and closely spaced radicals are prerequisites for energy storage applications, leading to an exchange narrowed EPR line. ORPs are instrumental in the pursuit of more sustainable energy storage technologies.^21^ They consist of pendant radical moieties as repeat units. These radical centers have unpaired electrons, which impart electrochemical activity to the polymer by undergoing redox reactions. The polymer structure used in this work, poly(2,2,6,6-tetramethylpiperidinyloxy-4-yl methacrylate) (PTMA), consists of 2,2,6,6-tetramethylpiperidinyloxyl (TEMPO) radicals as the redox unit with a methacrylate backbone. Owing to their electrochemical properties, ORPs find extensive use in emerging battery technologies such as organic radical batteries (ORBs).^22^ The redox properties can be tuned through chemical synthesis, enabling the construction of all-organic batteries,^23−25^ devoid of toxic metals. As the radicals cause paramagnetic properties, ORPs constitute a class of compounds that can be investigated using EPR.

EPR **g** tensors can be calculated at the DFT level.^26−29^ The **g** matrix is obtained through the calculation of three main contributions to the *g* shift consisting of a relativistic mass correction term (Δ*g*^RMC^),^30^ a diamagnetic gauge correction term (Δ*g*^DGC^),^31^ and a term related to orbital Zeeman and spin–orbit coupling interactions (Δ*g*^OZ/SOC^).^26,32^ The first two terms, Δ*g*^RMC^ and Δ*g*^DGC^, can be obtained from the spin density. At the DFT level, Δ*g*^OZ/SOC^ can be obtained through a solution of coupled-perturbed self-consistent field (CP-SCF) equations, which is also the approach used in the *ORCA* software package.^26,33^

We recently reported the use of the *g* value as a parameter for method validation of MD simulations of radical polymers.^34^ The evolution of *g* was monitored by calculating *g* using DFT for different time frames of the MD trajectory. Since *g* can be experimentally verified, characteristics of the simulated polymer can be tuned to match realistic sample conditions. For instance, experimental *g* can be compared with calculated *g* to judge the simulation time scales needed to obtain an equilibrated polymer structure. Furthermore, due to the sensitivity of *g* toward the molecular structure, features like the minimum chain length of the simulated polymer can be optimized.

DFT-based geometry optimization and **g**-tensor calculations on large disordered systems such as polymers are computationally demanding. While electronic structure methods that scale linearly with the number *N* of atoms in a system have been reported,^35,36^ computational costs of most DFT calculations scale with *N*^3^ for larger system sizes.^37,38^ In the case of **g**-tensor calculations, SCF procedures scale with *N*^2^.^26^ Adequate theoretical representations of radical polymers often require several monomers, which increase the system size (*N* > 200 for one polymer molecule), making the application of DFT methods less feasible. Additionally, experimental investigations of the cycling stability of ORBs indicate the importance of using cross-linked polymers, and, therefore, simulation systems need to include and account for additional cross-linking moieties, increasing the complexity of the simulated system further.^34^

Using *in operando* EPR techniques, evolution of the active material as a function of the state of charge can be studied.^39−41^ Here, the *g* value serves as a parameter that can be theoretically computed and experimentally verified to substantiate the required complexity of the simulated system and its similarity to the experimentally investigated states of charge. While simulation of battery systems with realistic complexity for various states of charge is attainable using MD, DFT implementations result in inferior scalability. Therefore, a computationally cheaper and scalable approach to predict *g* values for larger and more complex simulation systems would be desirable.

ML approaches provide an alternative to bypass the computational expense of electronic structure methods such as DFT. For instance, ML methods have been applied to learn density functionals itself, with the aim of avoiding the calculation of Kohn–Sham equations.^42^ Machine learnability of properties calculated using more accurate and computationally demanding electronic structure methods such as coupled cluster has also been reported.^43^ Learning algorithms that utilize neural networks usually require large data sets for training, and the learning process can be computationally expensive. In the case of size-limited data sets, ML algorithms such as regression trees, Gaussian process regression (GPR), support vector regression (SVR), or kernel ridge regression are more suitable.^44−46^ For instance, applicability of tree-based algorithms for p*K*_a_ predictions in proteins^47^ and GPR for atomistic properties^48,49^ has been successfully demonstrated.

A key advantage of complementing electronic structure methods with ML techniques is speeding up the computation of properties and not limiting their calculation to small-sized systems. Within the field of magnetic resonance, ML is an emerging tool with similar aims. In particular, applying ML for nuclear magnetic resonance (NMR) chemical shift predictions from molecular structures has gained widespread use.^9,50^ ML methods also find utility in EPR data analysis, mainly in the subfield of hyperfine spectroscopy^51^ and dipolar spectroscopy.^52^ More recently, neighborhood component analysis, an ML algorithm, was utilized to quantify the importance of structural parameters in determining electron–nuclear hyperfine interaction tensors.^53^ Although analogous to chemical shifts, to the best of our knowledge, ML approaches to predict EPR *g* values have not been attempted yet.

In this work, an ML model is developed to predict *g* values from molecular structures of PTMA. The model is trained on the value to be predicted, *g*_iso_, and the corresponding structural characteristics of the polymer as the features. The dynamic evolution of *g* along the MD trajectory, which is correlated to the underlying molecular structure, is exploited to train the model with the aim of reproducing the evolution of *g* for unknown radical densities or, alternatively, for different states of charge of an ORB. The performance of the model is evaluated on test data sets, and the dependence of model performance on different molecular descriptors is discussed. Finally, the trained model is applied to an unknown MD trajectory to test its ability to completely predict the evolution of *g* along the trajectory. The model is also studied with respect to the quality of interpolation to unknown radical densities. The importance of specific features that determine the predictions of the model is evaluated and discussed in correlation with the molecular structure of the polymer.

## Workflow

**Figure 1:**
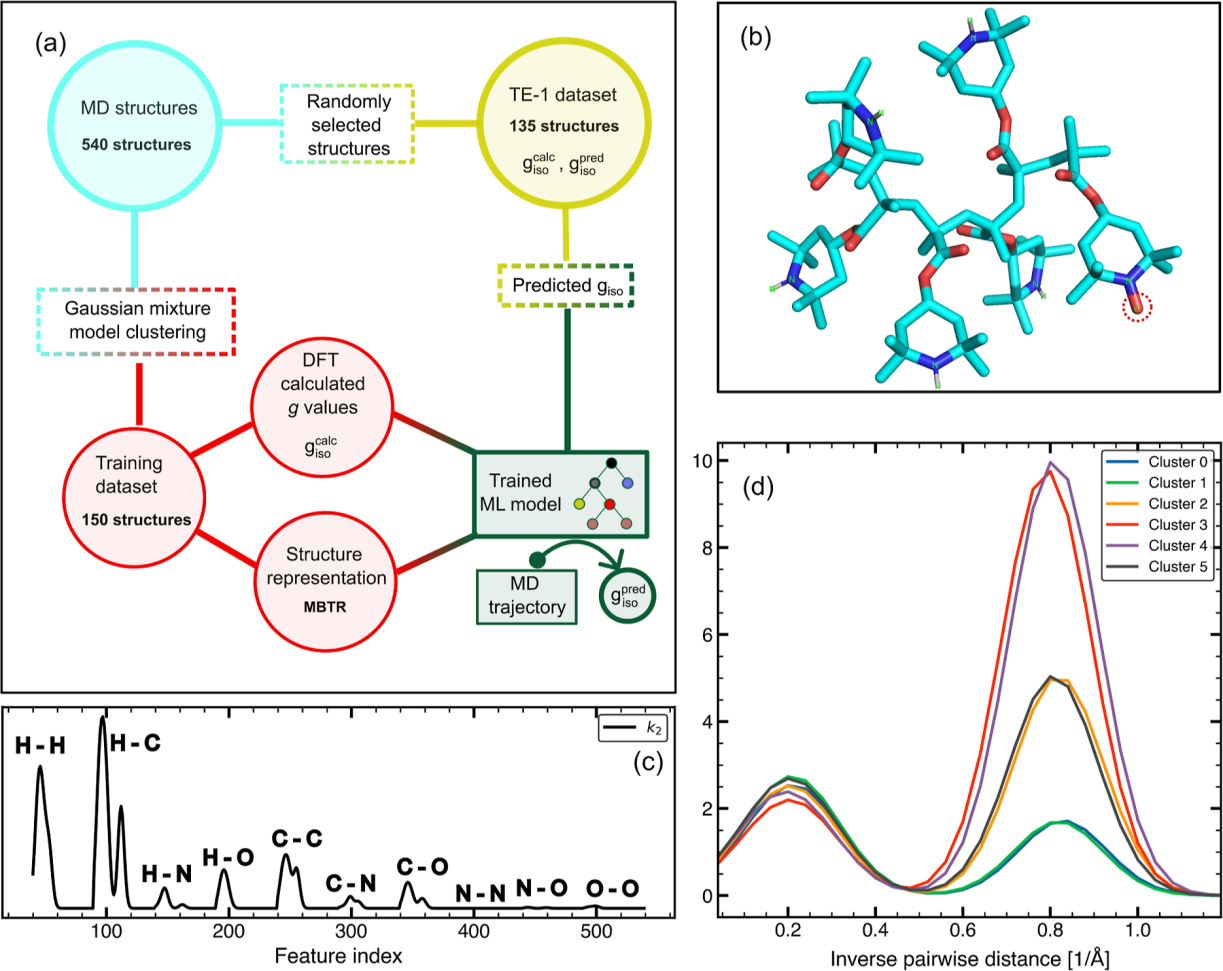
(a) Schematic representation of the workflow used for machine learning *g*_iso_. (b) Ball and stick model of PTMA-1, with six monomer units out of which one is a radical center (circled with a red dotted line). Oxygen atoms are colored red, nitrogen atoms are colored blue, and carbon atoms are colored cyan. (c) Schematic representation of the *k*_2_ term of MBTR for a PTMA-1 molecule. (d) Section of the N–O region of MBTR showing structural differences in cluster centers obtained using GMM. The distribution centered at an inverse pairwise distance of ≈0.8 Å^–1^ corresponds to the N–O bond length, and the distribution centered at ≈0.2 Å^–1^ corresponds to long-range N–O interactions. The distributions are weighted exponentially to give more weight to short-range interactions.

The workflow followed in this work is depicted in Figure 1a. First, PTMA polymer molecules are dynamically evolved in the presence of an electrolyte using classical MD (see “MD Simulations” in the Methods section) to simulate an organic cathode. For MD simulations, the polymer is represented by using a linear polymer structure made of six monomer units. Structures with different radical densities were generated by varying the number of monomers that are radicals. The notation used in this work is PTMA-*X*, where *X* denotes the number of monomers which are radicals. For instance, PTMA-6 represents 100% radical density, i.e., all six monomers are 2,2,6,6-tetramethylpiperidinyloxy-4-yl methacrylate (TEMPO methacrylate) radicals, while PTMA-1 corresponds to a structure where only one out of six monomers is a radical and the other five monomer units are diamagnetic 2,2,6,6-tetramethylpiperidin-4-yl methacrylate groups (see Figure 1b). From separate MD trajectories of PTMA-1, PTMA-3, and PTMA-6, polymer structures from different time frames (see “Data Set Generation” in the Methods section) are extracted to generate the whole structural data set (**WSD**).

For ML, input data need to be transformed into suitable features, which can be used to train the ML model. To predict structure-dependent properties, structural features of the molecule, such as interatomic distances and bond angles, can be utilized as features.^54^ To encode such features and represent the structure, the input data are transformed using a molecular descriptor. Molecular descriptors encode either a local atomic environment or the whole structure, depending on the property to be predicted. Local descriptors, such as smooth overlap of atomic positions (SOAP),^55,56^ encode local atomic environments, while global descriptors, such as many-body tensor representation (MBTR),^56,57^ encode the whole structure. The representations can be further tuned by user-defined and system-dependent parameters to capture only relevant atomistic interactions. In addition to the aforementioned descriptors, a third set of features, generated from bond lengths, bond angles, and dihedral angles [distances–angles–dihedrals (DAD)] extracted from the polymer structure, is also utilized for representing the molecular structure in this work. The parameterization and construction of molecular descriptors are described under “Molecular Representation” in the Methods section.

To ensure structural diversity in the training data set (**TR**), an optimum sampling of the configurational space of the polymer molecule is required. Clustering algorithms such as the Gaussian mixture model (GMM) clustering can be used to find clusters in the data set and sample configurationally diverse PTMA structures.^58−61^ GMM assumes that the data to be clustered originate from a mixture of multivariate Gaussian distributions with unknown means and covariances. GMM fits a specified number of distributions, which correspond to clusters in the data set and optimize the values of mean and covariance through the expectation–maximization algorithm.^62^ The cluster centers, or the mean of Gaussian distributions, are initialized randomly from structures in the data set itself and iteratively updated to maximize the likelihood of the data with respect to the distributions. To generate the **TR**, the atomic coordinates of structures in **WSD** are transformed using a suitable molecular descriptor. By clustering the structures in **WSD** multiple times with random initial clusters and selecting the structure at the center of each cluster on each run, the **TR** is obtained. To illustrate that this approach finds structurally diverse structures, the N–O distance regions of the MBTR output corresponding to six cluster centers from a single run of the clustering algorithm are shown in Figure 1d.

DFT calculations for obtaining *g*_iso_ need to be done only for the structures selected by the clustering algorithm, thereby reducing the computational cost of the ML workflow. From the remaining structures of **WSD**, which are not included in the **TR** data set, a subset of structures are randomly selected to generate a test data set **TE-1** for performance evaluation of the trained model. The structures in **TR** represented using a molecular descriptor and the corresponding DFT-derived *g*_iso_ (*g*_iso_^calc^) are used for training the model.

To select an appropriate learning method, various models trained using different types of learning algorithms were evaluated with **TR** using cross-validated scoring [see Section A1 and Figure S1 in the Supporting Information for a description of cross-validation]. The tested methods are chosen based on their known applicability to predict structure-dependent properties or for generating baseline models that warrant the need for more complex models. For instance, SVR, GPR, and tree-based ensemble methods have previously been demonstrated for prediction of properties such as NMR chemical shifts, which are analogous to *g* values.^9,63,64^ The performance of each trained model was then evaluated through cross-validated scoring with the **TR** data set represented using SOAP, MBTR, and DAD (see Section A2 in the Supporting Information). The model molecular descriptor pair with the best and most consistent predictive accuracy, based on the standard deviation of the error metric, is selected as the final model. The final model is then applied to structures derived from an unknown MD trajectory for further evaluation.

## Methods

### MD Simulations

For each of the three radical densities studied, PTMA-1, PTMA-3, and PTMA-6, the following classical MD simulation^65^ was used. Hereafter, this simulation is referred to as MD-1. Six monomers were used to represent each of the PTMA polymers and a total of 24 such polymers were used to mimic an organic electrode.^34^ As an electrolyte solution, 1064 ethylene carbonate (EC), 2100 ethyl methyl carbonate (EMC), and 300 LiPF_6_ molecules were chosen in a 10 × 10 × 10 nm^3^ simulation cell, with periodic boundaries along all three Cartesian coordinates. Partial charges for EC, EMC, and PF_6_^–^ were taken from the previous work.^66−69^ The initial configurations (*t* = 0) were constructed using PACKMOL,^70^ which avoids repulsive potentials by keeping a safe interatomic distance. A two-step NPT ensemble process was undertaken for all MD simulations using GROMACS 2019.^71^ Initially, a 2 ns initialization step with a 0.5 fs time step ensured system stabilization, where the Berendsen thermostat and barostat maintained the temperature at 298.15 K and pressure at 100 bar with 1.0 ps time constants.^65^ Subsequent to the initialization step, an equilibration step of 20 ns with a reduced 1 fs time step employed the Nosé–Hoover thermostat and Parrinello–Rahman barostat to regulate the temperature and reduce the reference pressure to 1 bar, keeping time constants constant at 1.0 ps. Throughout both steps, Coulombic and Lennard–Jones interactions were handled via a particle–particle-mesh solver with a consistent cutoff of 1.2 nm. The OPLS all-atom force field^72^ was used for all MD simulations. The atomic site charges on paramagnetic and diamagnetic repeating units of PTMA were calculated from the electrostatic potential (ESP) fit (see Figure S5 in the Supporting Information). Gaussian16^73^ was used to calculate the ESP charges using the MP2 theory^74^ with a pVDZ basis set.^75^ The structures from the MD-1 trajectory were used for training the model (see the “Data Set Generation” section).

For additional model evaluation, separate MD simulations (MD-2), with only one polymer chain in the simulation box, were used for generating PTMA-1, PTMA-2, PTMA-3, PTMA-4, and PTMA-6 structures. Other MD parameters were kept the same as for MD-1. The structures from MD-2 were solely used for generating test data sets (see the “Data Set Generation” section).

### DFT Calculations

DFT computations of *g* values were conducted using ORCAv. 5.0.2^76^ and PTMA polymer structures obtained from different time frames of the MD trajectory. The **g**-tensor origin was set to the center of spin density and calculations were done using the unrestricted Kohn–Sham formalism, with the B3LYP functional and EPR-II^77^ basis set. Automatic generation of auxiliary basis sets was used for all calculations.^78^ Typical calculation times ranged from 1 to 3 h per structure while running in parallel on 12 cores. Optimization of the DFT calculation protocol for TEMPO methacrylate and PTMA is described elsewhere.^34^

### Data Set Generation

To generate the **WSD**, a total of 540 structures were sourced from separate MD trajectories of PTMA-1, PTMA-3, and PTMA-6. From the initialization step of MD-1, seven logarithmically spaced time frames were sampled from 0.0005 to 500 ps. From the equilibration step of MD-1 ranging from 2000 to 20,000 ps, structures were sampled linearly in steps of 2000 ps. As structures from the same time frame show variation in the DFT-derived *g*_iso_ (*g*_iso_^calc^), a total of 10 structures per time frame were extracted. In addition, 10 structures for each radical density from the start of the MD trajectory (*t* = 0) were also added to **WSD**. The number of PTMA-1, PTMA-3, and PTMA-6 structures is equal in **WSD**. To generate the **TR**, GMM as implemented in scikit-learn v. 1.2.2^79^ was applied to structures in **WSD**, with each structure represented using a molecular descriptor (see the “Molecular Representation” section). For GMM, initialization of clusters was done randomly from the data set. Upon convergence of the clustering algorithm, the structure at the center of each cluster (or the mean of each Gaussian distribution) was added to the **TR**. Due to the randomness in selecting the initial clusters, 50 runs of the algorithm were done to obtain 150 unique structures that form the **TR**. The test data set **TE-1** was generated by randomly selecting 135 structures from the remaining structures of **WSD**. Another test data set **TE-2** was generated by sourcing structures from the MD trajectory of MD-2, consisting of equal numbers of PTMA-1, PTMA-2, PTMA-3, PTMA-4, and PTMA-6 structures.

### Molecular Representation

Chemical structures in the XYZ file format were read using the Python library atomic simulation environment (ASE) v. 3.22.1.^80^ For implementation of SOAP^55^ and MBTR,^54^ the DScribe library^56^ (v. 1.2.2) was used. In the SOAP formalism, atomic positions were represented by using Gaussian functions. This allows for structural representation in terms of atomic neighbor density around a central atom given by
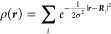
1where ***r*** is the position in the space of the central atom, ***R***_*i*_ is the position of a neighboring atom, and summation *i* runs over all atoms within a specified cutoff distance from the central atom. The atomic density was calculated for each atom in the molecular structure and was expanded using a combination of spherical harmonics and radial basis functions (RBFs).^55^ The final SOAP output was constructed as a power spectrum of the expanded atomic density.^56^

In the MBTR, a geometry function was used to transform a configuration of *k* atoms into a single value. Commonly used geometric functions for *k* = 1, *k* = 2, and *k* = 3 configurations corresponded to atomic numbers (*Z*), inverse pairwise distances , and angles between three atoms ∠(***R***_1_ – ***R***_2_, ***R***_3_ – ***R***_2_), respectively. The values obtained from the geometric functions were then converted into distributions using the Gaussian kernel density estimation. The final MBTR output consisted of concatenated Gaussian distributions corresponding to all possible combinations of *k* atoms and was weighted according to the number of occurrences of a particular combination in the molecular structure.^56^ As an example, Figure 1c shows the *k*_2_ term of the MBTR output for a molecule of PTMA-1 (see Figure 1b), depicting the inverse distances between atom pairs of the PTMA oligomer.

In the case of SOAP and MBTR, cutoff distances (see Table S4) and number of features (see Table S5) were optimized using the **TR** data set and cross-validated scoring (see Section A6 in the Supporting Information). Parameters for feature vectors used to train the final model are described below. In the case of SOAP, the cutoff distances were set to 10 Å, and spherical Gaussian-type orbitals were used as RBFs. The number of RBFs and maximum degree of spherical harmonics was set to 8. For MBTR, geometric functions for *k*_1_, *k*_2_, and *k*_3_ terms corresponded to atomic numbers, inverse pairwise distances in units of Å^–1^, and angles between three atoms in degrees, respectively. The bounds for the *k*_1_ term were set to 0 and 10 with the number of discretization points set to 10. For *k*_2_, a distance range of 0.5–25 Å (inverse distance range of 0.04–2 Å^–1^) was used and the number of discretization points was set to 50. For the *k*_3_ term, the number of discretization points was set to 180. In the case of *k*_2_ and *k*_3_, the distributions were weighted exponentially as a function of distance to give more importance to structural properties corresponding to closely spaced atoms. A distance cutoff of 10 and 5 Å was used for weighting the *k*_2_ and *k*_3_ terms, respectively. The Gaussian smoothing width (σ) was set to 0.01 for the *k*_1_ term, 0.1 for the *k*_2_ term, and 4 for the *k*_3_ term. The DAD molecular representation was built using bond lengths (Å) between two atoms, bond angles (°) made by three atoms, and dihedral angles (°) between two atomic planes. Bond lengths, angles, and dihedral angles were extracted using ASE. For all structural features, only bonded atoms within a cutoff distance of 1.5 Å were considered. The values were sorted according to the respective atomic combinations and concatenated to form the final DAD feature vector.

### Model Building

The ML algorithm for the final model was selected using cross-validated scoring with the **TR** data set (see Section A2 and Figure S2 in the Supporting Information). The main types of considered methods were SVR,^81^ GPR,^9,48^ regression trees,^82^ ensemble methods, and linear regression. In the case of ensemble methods, averaging^44,83^ and boosting^84,85^ techniques were tested.

The final model based on regression trees (see Section A3 in the Supporting Information for a theoretical description), which is used in this work, was built as follows. A regression model based on the extremely randomized trees (ERT) method^46^ was generated using scikit-learn v. 1.2.2^79^ (ExtraTreesRegressor in *scikit-learn*). The model was trained on the **TR** data set. Parameters that affect the learning process, known as hyperparameters, were optimized through a bound constrained and exhaustive grid search (GridSearchCV in *scikit-learn*). The root mean squared error (RMSE) was minimized during the optimization. The grid search bounds and optimized hyperparameters are listed in Table S1 in the Supporting Information. Hyperparameter optimization was also attempted using Optuna^86^ (see Section A4 in the Supporting Information) and the optimized parameters are listed in Table S2. For the final model, parameters obtained from the grid search were used, based on the performance metrics (see Table S3). The ERT model was always initialized with a random state of 1 for reproducibility. The performance of the model on the test data sets TE-1 and TE-2 was quantified using the coefficient of determination (*R*^2^), mean absolute error (MAE), and RMSE. A mathematical description of the error metrics is given in Section A5 of the Supporting Information. The training time of the final model using the **TR** data set (represented using MBTR) with the optimized hyperparameters was 4.97 ± 16.6 ms while running on a single core (Apple M1 processor) and 926 ± 13.7 ms when parallelized on 8 cores (Apple M1 processor). The time required for prediction was 3.58 ms ± 67.2 μs (single core). The reported times are mean ± standard deviation of 7 runs.

## Results and Discussion

### Evolution of Calculated *g*_iso_

**Figure 2:**
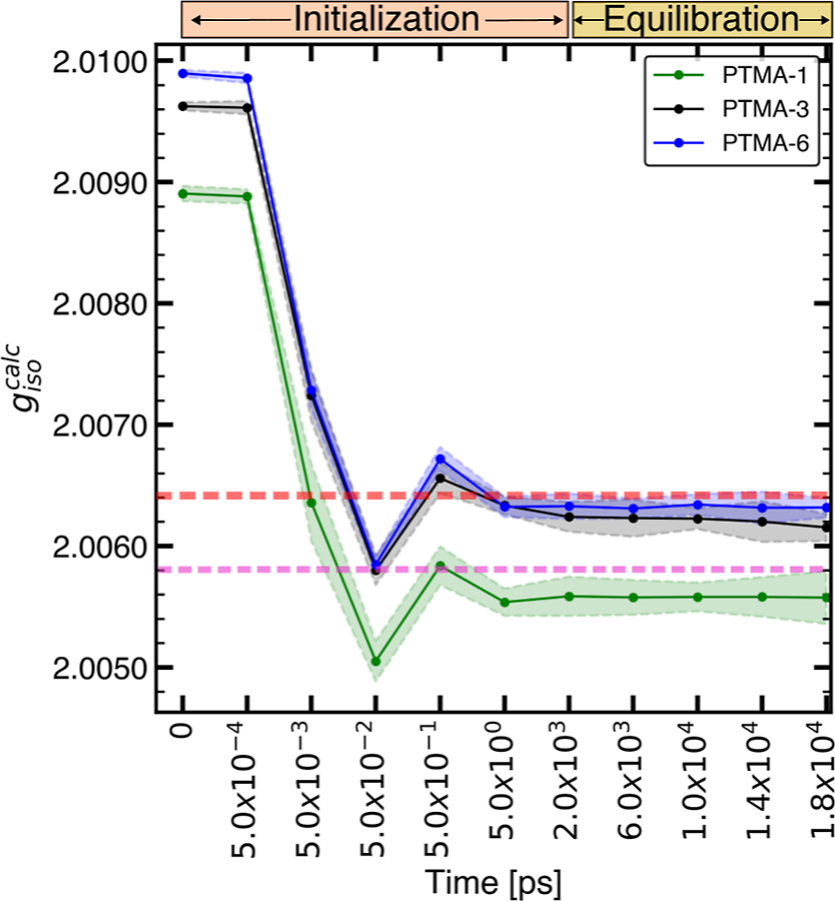
Evolution of *g*_iso_^calc^ along the MD-1 trajectory. Each data point is the average of *g*_iso_^calc^ for 10 individual structures. Shaded regions denote the standard deviation. Before the equilibration run starting at 2000 ps, an initialization step was done. Experimental *g*_iso_ for PTMA (red) and its monomer (magenta) in the solution state equal to 2.0064 and 2.0058, respectively, and are denoted by dashed lines.

Figure 2 shows the DFT-calculated *g* values (*g*_iso_^calc^) for polymer structures extracted from different time frames of the MD trajectory. Each data point represents the mean  of *g*_iso_^calc^ of 10 randomly selected structures for a specific time frame. The structures differ in their radical density, i.e., the number of monomer units that are radicals. The experimental analogue for the PTMA-6 (100% radical density) is a PTMA polymer in a state of high radical density, such that radical–radical interactions are significant. In the case of PTMA-1,  is compared to the experimental *g* value obtained from a dilute solution of TEMPO methacrylate (PTMA monomer), with negligible radical–radical interactions. As an additional experimental reference, experimental *g* values of PTMA for transient states of charge are obtained from the *in operando* EPR measurement of an ORB with PTMA as the active material (see Section A10 in the Supporting Information). The *in operando* EPR experiments show that changes in *g* are reversible during battery cycling and are correlated to the radical density (see Figure S6a,b in the Supporting Information).

Experimental *g* values (*g*_iso_^exp^) of the PTMA polymer and TEMPO-methacrylate solutions were reported to be 2.0064 (dashed red line in Figure 2) and 2.0058 (dashed magenta line in Figure 2).^34^ For a pristine ORB, *g*_iso_^exp^ of PTMA was found to be 2.0065 (see Figure S6a in the Supporting Information). For PTMA-6, *g*_iso_^calc^ is in good agreement with both corresponding experimental references, with  being 0.0001 in the case of the polymer solution and 0.0002 in the case of PTMA with high radical density in the ORB. At an intermediate radical density of PTMA in the ORB, *g*_iso_^exp^ already approaches values which are predicted by DFT for PTMA-1, a simulated structure with low radical density. The comparison of *g*_iso_^calc^ corresponding to PTMA-1 with *g*_iso_^exp^ of a dilute solution of TEMPO methacrylate shows a difference of 0.0003.

For both experimental references, a better agreement between *g*_iso_^exp^ and *g*_iso_^calc^ is observed at high radical density in comparison to that at low radical density. The larger deviation between *g*_iso_^exp^ at lower radical density and *g*_iso_^calc^ of PTMA-1 may be explained by considering the structural and conformational differences between the experimental and simulated structures. A dilute solution of nitroxide radicals does not include the structural effects of the extended oligomer chain. In the simulated structure of PTMA-1, the radical species is restricted to the oligomer backbone, while in a dilute solution of TEMPO methacrylate, the radical undergoes isotropic motion. In the case of PTMA in an ORB, the disagreement between *g*_iso_^calc^ of PTMA-1 and *g*_iso_^exp^ for a state of low radical density of the ORB may stem from interactions between PTMA and other battery constituents, which are more effective when intramolecular radical–radical interactions are negligible. It is known that conductive additives used in organic cathodes affect experimental EPR parameters and battery performance.^87^ However, the current complexity of the simulated system does not account for such interactions. Another reason could be long-range radical–radical interactions which are not captured in the DFT calculations with individual polymer chains but are expected to be present even at low and intermediate radical densities as the polymer prefers to maximize radical–radical interactions.^34,88,89^ On the other hand, the better agreement at higher radical densities also indicates that at high radical density, for any particular redox unit, radical–radical interactions are adequately represented by the simulated structure consisting of only radicals. Inclusion of multiple, longer polymer chains and modeling of conductive additives in the simulated system may improve the agreement also for low and intermediate radical densities. However, EPR parameters of such complex systems at transient states of charge might not be accessible by DFT methods due to the high computational cost.

The PTMA structure at time *t* = 0 for each radical density corresponds to the structure before equilibrium and shows a large deviation from *g*_iso_^exp^. However, the extent of deviation seems to be dependent on the radical density, providing a measurable *g* value difference between the structures before equilibrium for each radical density. Furthermore, the structure at *t* = 0 serves as an important evaluation criterion for the ML model to assess its ability to differentiate between radical densities. Timeframes up to 2000 ps correspond to the initialization step. A rapid convergence of  to *g*_iso_^exp^ is observed as the simulated system stabilizes. Changes in  are minimal beyond 50 ps, indicating an equilibrated system.

DFT-derived *g*_iso_^calc^ values allow for a differentiation of the polymer structures through two aspects. First, structures before equilibrium and structures after equilibrium show significant differences in *g*_iso_^calc^, and second, *g*_iso_^calc^ changes with respect to the radical density. For an ML model to be a viable alternative, the model should also learn to differentiate polymer structures based on the two aforementioned aspects. Additionally, the evolution of *g*_iso_^calc^ along the MD trajectory can only be reproduced if the model learns to correlate the magnitude of change in *g*_iso_^calc^ to the extent of structural changes. Despite structural differences, PTMA-3 and PTMA-6 showed small differences in converged values for , while a larger contrast is obtained when PTMA-1 is compared to PTMA-3 and PTMA-6.

By including PTMA-3 in the **TR**, the trained model can be expected to learn smaller *g*_iso_ changes or equivalently subtle structural deviations, which occur as a result of a change in the radical density. This should allow for more precise *g*_iso_ interpolations for radical densities that lie within the limits of **TR** but are not included in the **TR**.

### Performance Evaluation and the Analysis of Regression Tree Models

Among the tested ML methods, ensemble methods which use a combination of multiple models showed the best performance with mean deviations of *g*_iso_^calc^ from predicted *g*_iso_ (*g*_iso_^pred^) ranging from 0.0001 to 0.0004 (see Figure S2 in the Supporting Information). In comparison, kernel-based methods, GPR and SVR, showed larger mean deviations ranging from 0.0002 to 0.0006. Overall, models trained on MBTR feature vectors showed better predictive accuracy in comparison to SOAP and DAD feature vectors. Through cross-validation, the spread of MAE and RMSE scores also suggests that the models trained on MBTR perform consistently better on different folds of the **TR** data set. With MBTR feature vectors, even simpler models such as linear regression show RMSE values of the order of 0.0004, while the model performs considerably worse in the case of DAD. This is likely due to the nonlinear mapping of structural properties in the case of MBTR, which was also observed previously for ML-based predictions of exchange spin coupling.^49^ In DAD, the values of angles and distances are used without further transformation, and a linear relationship between the structural properties and *g*_iso_ may not be present. However, in the case of kernel-based methods and ensemble methods, which can handle nonlinear data, models trained on DAD show improved performance. A combination of ERT and MBTR showed the best performance with the lowest MAE and RMSE values (see Section A2 in the Supporting Information).

Table 1 compares the cross-validated error metrics for the ERT model in the case of three different molecular descriptors investigated in this work. ERT models trained on global descriptors perform considerably better than in the case of the ERT model trained on SOAP feature vectors. This indicates that in the case of the polymer structure, the property to be learned, *g*_iso_, is affected by the structural features of the whole structure rather than a localized region. This observation is in agreement with CW-EPR experiments using PTMA, where polymer and monomer samples show variations in the observed *g* value. Furthermore, as shown in the previous section, DFT calculations using polymer structures with different radical densities also show structure-dependent changes in *g*_iso_^calc^. *In operando* EPR results (see Section A10 in the Supporting Information) further confirm that the *g*_iso_ value of the active material, PTMA, is influenced by the change in the radical density (see Figure S6). As changes in the radical density can be accompanied by changes in polymer conformation,^34,88,89^ descriptors which encode the global polymer structure, such as MBTR and DAD, fare better in *g* value predictions. The performance of the ERT model was also studied with respect to the cutoff distance used for SOAP and MBTR. For SOAP, a cutoff distance of 10 Å was found to be optimal. In the case of MBTR, slight improvements in RMSE were observed with larger cutoff distances (see Section A6 in the Supporting Information). A cutoff distance of 10 Å was used for both SOAP and MBTR based on the RMSE (see Table S4). As the distance between two adjacent monomer units on the oligomer chain lies in the range of 7–10 Å, a cutoff distance of 10 Å should capture most of the intramolecular interactions between the monomer units.

**Table 1:** Cross-Validated Performance Metrics Obtained for Different Molecular Descriptors Using the **TR** Data Set

molecular descriptor	*R*^2^	MAE [×10^–4^]	RMSE [×10^–4^]
SOAP	0.87 (0.085)	1.91 (0.37)	2.59 (0.68)
MBTR	0.95 (0.019)	1.25 (0.14)	1.58 (0.20)
DAD	0.92 (0.034)	1.53 (0.25)	2.07 (0.45)

Among the two global descriptors, the performance was better for the model trained on MBTR feature vectors. While DAD also encodes the whole structure in the form of bond lengths, bond angles, and dihedral angles, MBTR likely captures long-range interactions of adjacent radical units better due to the larger cutoff distance. This aspect becomes especially important when interpolating predictions for different radical densities, where the presence or absence of a neighboring radical moiety affects *g*_iso_^calc^. However, prompted by similar performance metrics, ERT models trained on both MBTR and DAD were evaluated using the TE-1 test data set.

**Figure 3:**
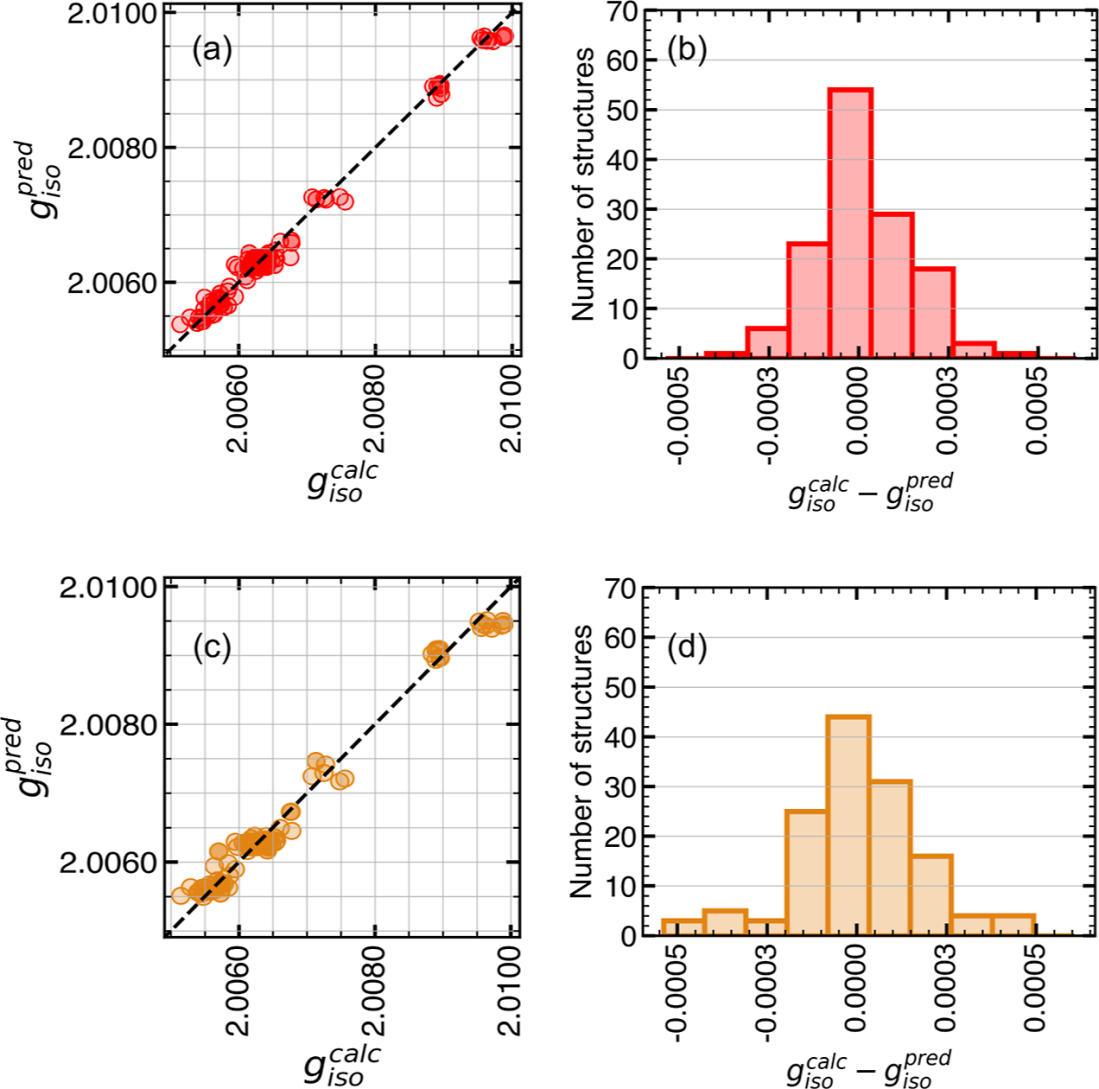
Performance evaluation of the model trained on MBTR and DAD structures using the TE-1 data set. Correlation plot showing *g*_iso_^calc^ vs *g*_iso_^pred^ for TE-1 structures represented using (a) MBTR and (c) DAD descriptors. Histograms showing *g*_iso_^calc^ – *g*_iso_^pred^ for TE-1 structures represented using (b) MBTR and (d) DAD descriptors.

Figure 3a–d summarizes the dependence of model performance on MBTR and DAD. Hereafter, the ERT models trained on MBTR and DAD features are referred to as ERT-MBTR and ERT-DAD, respectively. The error metrics given in Table 2 indicate 27% (based on MAE) better performance for ERT-MBTR. In comparison to ERT-DAD, a narrower spread (see Figure 3b) of *g*_iso_^calc^ – *g*_iso_^pred^ and a lower RMSE for ERT-MBTR suggest that the predictions do not deviate heavily from *g*_iso_^calc^. ERT-MBTR shows superior prediction accuracy especially in the *g* value range of 2.0090–2.0100 (see Figure 3a) when compared to ERT-DAD (see Figure 3c). The structures in this range, which show a significant deviation from the experimental *g* values, are obtained from the initialization step before the start of the equilibration step (*t* = 2000 ps). The ability of the model to differentiate between structures before equilibrium and equilibrated structures is essential for application to new MD trajectories. For equilibrated structures in the case of PTMA-6 and PTMA-3, *g*_iso_^calc^ lies in the range of 2.0060–2.0066. As differences between structures are minimal in this regime, predictions in agreement with *g*_iso_^calc^ require the model to be sensitive to minor changes in the structural features. ERT-MBTR seems be more sensitive to such changes, evident from the better performance compared to ERT-DAD in the corresponding *g* value range. Model performance of ERT-MBTR remained similar toward all radical densities in **TE-1**. Similar *R*^2^ scores of 0.990, 0.986, and 0.982 were obtained for evaluation using PTMA-1, PTMA-3, and PTMA-6 structures, respectively, indicating that the model was not overfitting features from a specific radical density.

**Table 2:** Error Metrics Obtained for Different Molecular Descriptors Using the **TE-1** Data Set

molecular descriptor	*R*^2^	MAE [×10^–4^]	RMSE [×10^–4^]
MBTR	0.989	0.97	1.27
DAD	0.979	1.28	1.72

**Figure 4:**
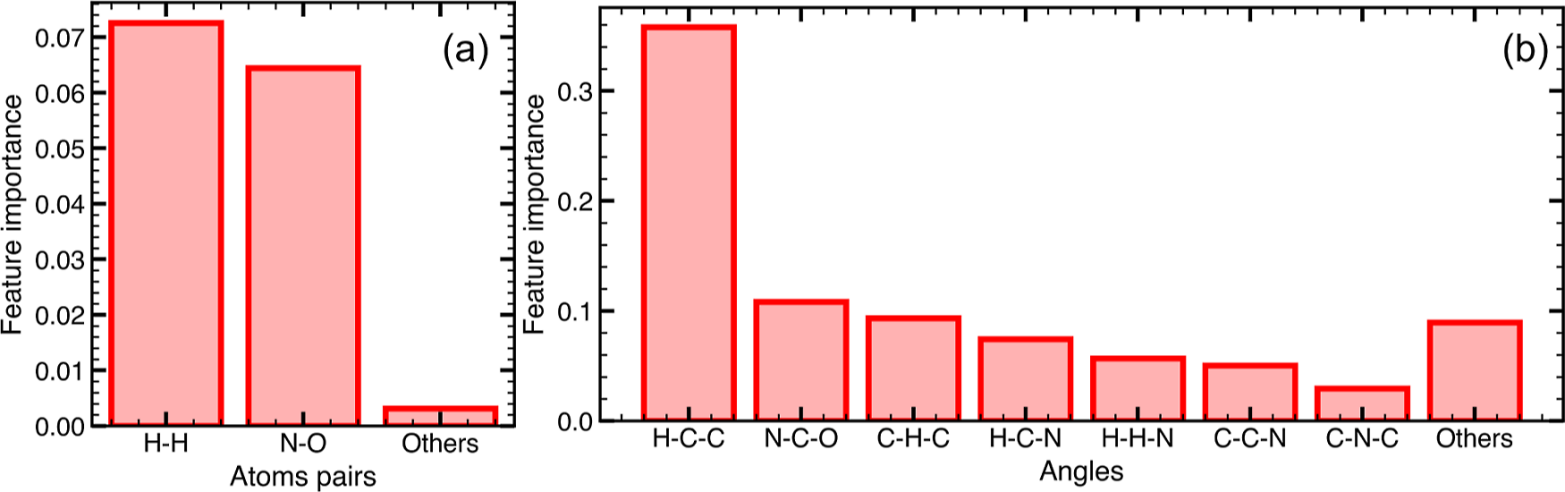
Feature importance scores for ERT-MBTR for (a) distance features and (b) angle features. Angles in “Others” have importance scores less than 0.027.

To interpret ERT models further, feature importance scores were analyzed. For simplicity, the importance of each structural feature was summed over all discretization steps for the feature under consideration. For ERT-MBTR, among features which encode pairwise distances, N–O and H–H were found to be important (see Figure 4a).

Dependence on N–O can be rationalized, as PTMA-1, PTMA-3, and PTMA-6 directly differ in the number of N–O bonds. Since MBTR feature vectors employ Gaussian distributions weighted according to the number of occurrences of a particular feature, PTMA-1, PTMA-3, and PTMA-6 with 1, 3, and 6 N–O bonds can be differentiated. However, a similar importance toward the N–H bond, which serves as an equivalent feature to differentiate between PTMA-1, PTMA-3, and PTMA-6, was not found. Note that further dependence on the N–O feature may arise from through-space interactions within the cutoff distance used for MBTR, for instance, between the nitrogen atom and oxygen atoms of the methacrylate backbone (see Figure 1b). An inspection of the MBTR output for the N–O feature in PTMA-1 (see Figure 1d) shows two distributions centered at ≈1.3 Å (0.8 Å^–1^) and ≈5 Å (0.2 Å^–1^). The shorter distance corresponds to the N–O bond length of TEMPO methacrylate radicals. The longer distance corresponds to the distance between the N atom and the carbonyl O atom of the methacrylate branch. For PTMA-6 and PTMA-3, interactions between N atoms and O atoms on adjacent monomer units may also contribute to the importance of the N–O feature. The N–O distance was also found to be an important distance feature in the case of ERT-DAD, along with N–H (see Section A7 and Figure S3a in the Supporting Information). There are significantly more H–H interaction pairs within the cutoff distance than N–O interactions; therefore, pinpointing specific interactions in the case of H–H is challenging. Among features which encode angles formed by three atoms, H–C–C and N–C–O angles were found to be important for ERT-MBTR (see Figure S4b in the Supporting Information). The importance of H–C–C and N–C–O features is possibly related to the conformational changes in the six-membered ring of the radical moiety. This suggestion is also supported by the observation that C–N–O angles as well as C–C–C–C and C–N–C–C dihedral angles are important features in the case of ERT-DAD (see Figure S3b,c in the Supporting Information). Features in the *k*_1_ dimension of MBTR, i.e., atomic numbers, were not found to be important in comparison to *k*_2_ and *k*_3_ features, possibly due to the **TR** containing molecular structures made up of the same type of atoms. However, these features might become important if a model is trained on data containing different radical moieties, differing in their type of atomic species. For both ERT models, while the most important features remained the same, their relative importance was found to change with the shuffling of **TR** and initializing the learning algorithm with different random states. Moreover, feature importance scores may be biased toward features with high cardinality or a higher number of unique values in comparison to other feature types (see Section A3 in the Supporting Information).^44,45,90^ For instance, as element H has the highest number of atoms in PTMA and long-range interactions are included in MBTR, possible values of H–H distances as a feature can take multiple, unique values, leading to a larger possibility of splits using this feature and a biased importance score. Therefore, only a qualitative discussion of features with consistently high importance scores was done, and a quantitative comparison of different features based on feature importance scores was not attempted.

To summarize this section, the ERT model trained on MBTR feature vectors affords predictions of *g*_iso_ with mean deviations from *g*_iso_^calc^ of the order of 0.0001 which is comparable to the magnitude of variation in *g*_iso_^calc^ per time frame. The obtained errors are also comparable to experimentally accessible *g* shifts, which are usually of the order of 10^–4^. Furthermore, the model was found to be sensitive to the radical density, as well as to the extent of equilibration.

### Application to MD-2

The final ERT-MBTR model was applied to structures from MD-2 to evaluate its ability to reproduce the evolution of *g*_iso_ along unknown MD trajectories. MD-2 differs from MD-1 in terms of the number of oligomer molecules used in the simulation. MD-2 with each PTMA-*X* (*X* = 1, 2, 3, 4, and 6) consists of only one oligomer molecule in the simulation cell instead of 24 in the case of MD-1. Therefore, the oligomer evolves under the influence of only intramolecular interactions and its interactions with the electrolyte. Furthermore, the model is evaluated on oligomer molecules that are structurally dissimilar from structures in **TR** in terms of the number of monomer units that are radicals. For this purpose, two unknown radical densities, PTMA-2 and PTMA-4, with one less and one more radical unit, respectively, in comparison to PTMA-3 were selected. For **TE-2**, the structures are extracted only from the initialization step of each PTMA-*X* MD trajectory, as larger *g* deviations occur only during the initial time frames of the MD trajectory, and *g*_iso_^calc^ was found to converge already during the initialization step.

**Figure 5:**
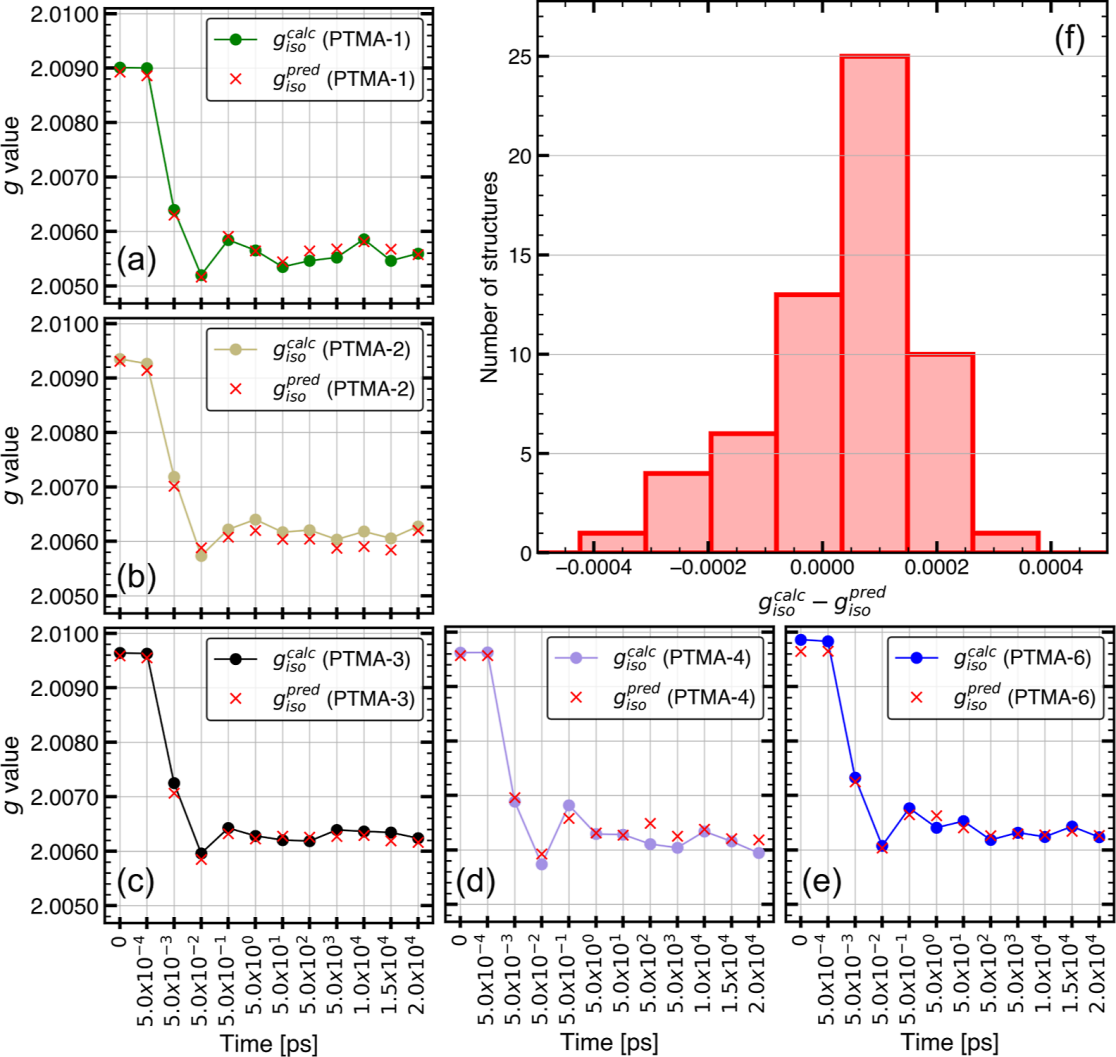
Prediction of *g*_iso_ from structures in the TE-2 data set. (a) Comparison of *g*_iso_^pred^ with *g*_iso_^calc^ for TE-2 structures with radical densities of (a) PTMA-1, (b) PTMA-2, (c) PTMA-3, (d) PTMA-4, and (e) PTMA-6. (f) Histogram showing *g*_iso_^calc^ – *g*_iso_^pred^ for structures in TE-2.

Figure 5 summarizes the predictive ability of the model for structures in **TE-2**. For PTMA-1, PTMA-3, and PTMA-6, the evolution of *g*_iso_^pred^ is in excellent agreement with *g*_iso_^calc^ (see Figure 5a,c,e). While comparatively larger mean deviations were observed for PTMA-2 and PTMA-4 (see Figure 5b,d), the evolution of *g*_iso_ is fairly well reproduced and MAE/RMSE values did not exceed 0.0002 (see Table 3) for any radical density present in **TE-2**. For the whole **TE-2** data set, the order of deviation of *g*_iso_^pred^ from *g*_iso_^calc^ (see Figure 5f) is similar to the deviations observed for **TE-1** with both MAE and RMSE values of the order of 0.0001 (see Figure 3b,d). This indicates that the ERT-MBTR model generalizes well toward unknown MD trajectories. Additionally, for each radical density in **TE-2**, the model effectively differentiates between the structures at the beginning of the simulation and the equilibrated structures once the convergence of *g*_iso_^calc^ is achieved.

**Table 3:** Error Metrics Obtained for the ERT-MBTR Model in the Case of the **TE-2** Data Set and Individual Radical Densities

data set	*R*^2^	MAE [×10^–4^]	RMSE [×10^–4^]
TE-2	0.989	1.15	1.38
PTMA-1	0.992	0.95	1.14
PTMA-2	0.981	1.54	1.65
PTMA-3	0.993	0.95	1.02
PTMA-4	0.982	1.31	1.73
PTMA-6	0.991	1.02	1.23

Better predictive performance toward PTMA-1, PTMA-3, and PTMA-6 is expected due to the composition of the **TR** data set used for training, which consists of these radical densities. However, the ability of the model to interpolate to other radical densities that are not included in the **TR** data set is found to be sufficient for the application. The largest MAE was obtained for PTMA-2, which did not exceed 2 × 10^–4^ (see Table 3). In comparison, MAE values are slightly lower for PTMA-4 but RMSE indicates deviations that are larger in magnitude. In both cases, deviations seem to be more prominent after the convergence of *g*_iso_^calc^ occurs, or equivalently, after the system reaches equilibrium and only minor variations in structural features occur. A comparison of MBTR outputs of structures from different time frames reveals that while structures before equilibrium are considerably different from equilibrated structures, only minor differences in the structural features are observed after *g*_iso_^calc^ converges (see Figure S4 in the Supporting Information). Nevertheless, the errors are smaller than the experimental *g* shifts observed for PTMA and comparable to environment-dependent *g* shifts measurable by high-field EPR in the case of organic radicals. The ability of the model to differentiate between equilibrated structures indicates that subtle structural changes are identified by the model, and consequently, minor differences in *g*_iso_^calc^ after convergence is reached are also reproduced well. Therefore, in addition to predictive accuracy with respect to absolute *g*_iso_ values, *g* shifts occurring for smaller changes in the state of charge can also be targeted. More fine-grained states of charge are still feasible through MD, and in this case, the ML approach serves as a more scalable method than DFT to predict *g* shifts for a larger set of structures.

## Conclusions

The applicability of ML methods to predict *g*_iso_, an EPR observable, from the structural features of PTMA, an organic radical polymer, was examined. PTMA molecules were dynamically evolved in an electrolyte using classical MD simulations, and oligomer structures were derived from the MD trajectory. Molecular descriptors, which encode either local or global structural features, were used to represent the molecular structure. A model based on regression trees was trained and the dependence of model performance on the encoding of structural features was studied. In the case of PTMA, global molecular descriptors such as MBTR, which encode the whole structure, were found to be more suitable. Mean deviations of ML-predicted *g* values (*g*_iso_^pred^) from DFT-calculated *g* values (*g*_iso_^calc^) were of the order of 1 × 10^–4^, which is accurate enough to detect structure- and environment-dependent *g* shifts in the case of PTMA. Furthermore, the evolution of *g*_iso_^pred^ along an unknown MD trajectory showed remarkable agreement with the evolution of *g*_iso_^calc^, thereby making the proposed ML-based approach a viable method to predict *g* and validate MD protocols for PTMA and similar systems. As the total computational cost of training and prediction using the ML model is lower than DFT, the approach offers a method with better scalability to predict *g*_iso_. Consequently, *g*_iso_ shifts between transient states of the radical polymer observed experimentally using EPR and simulated using MD can be compared. Since the agreement between *g*_iso_^pred^ and experimental *g* values is tied to the data used for training, MD simulations of the ORB system need to account for complex active material environments. As polymer conformations may be affected by interactions with other polymer chains, *g* value variations may also be influenced by interchain effects. A better representation of the experimental system may be achieved by the inclusion of multiple polymer chains and other electrode constituents such as conductive additives. In this aspect, the presented protocol can be utilized to compare MD protocols and test for agreement with an experimental system by using *g* as an experimental observable. A further refinement of the simulated system based on insights from EPR experiments can improve the agreement between *g*_iso_^calc^ and experimental *g* values also for low radical densities, which, in turn, should afford *g*_iso_^pred^ consistent with experimental g values. As the variation in *g*_iso_ with the radical density is larger than the error of the ML model, experimental *g* values can be used to benchmark such a combination of MD and ML. The protocol used in this work can be extended to larger simulation systems by conducting predictions on smaller subsystems and obtaining a distribution of *g* values for further statistical analysis and validation of large-scale MD simulations. Furthermore, the workflow can be transferred to other paramagnetic species provided that the *g* values can be experimentally verified and theoretically computed with sufficient accuracy. While isotropic parameters and their distributions can be targeted using the current protocol, transferability to tensorial properties may need to be investigated separately.

The ML workflow utilized in this work prioritizes low computational costs and uses a limited number of structures for training. While further gains in prediction accuracy may be achieved by increasing the size of the **TR**, the obtained accuracy is in line with the aims of the work. Upon application to unknown structures, the predictive accuracy was found to be higher for radical densities included in the **TR**s. Therefore, performance gains may be achieved by including additional radical densities in the **TR**. In order to extend the scope of the model, data-efficient protocols must be developed. Through an optimization of the **TR**, the approach can be extended to MD simulations which may vary from the simulations shown in the current work, in terms of simulation parameters such as temperature, chain length of the polymer chain, or radical moieties. To realize such functional extensibility, the ML workflow demonstrated in this work can be adapted into an active-learning ML workflow in which structures sourced from MD trajectories are continuously screened with respect to their dissimilarity from the current **TR** so that dissimilar structures can be added to a modified **TR** and used to retrain the model. Notably, we envision that the workflow can be adapted to other redox-active organic materials and that other properties relevant to organic radical polymer batteries, such as electron coupling parameters, may be targeted. As the predicted *g* values remain sensitive to the radical density and structural changes, the state of charge-dependent *g* shifts for ORB setups may become computationally accessible and may be applied to even larger simulation systems.

## Data Availability

Data sets, trained models, and scripts used in this work are available at Jülich DATA (https://doi.org/10.26165/JUELICH-DATA/TOBXWP) and Jülich Gitlab (https://jugit.fz-juelich.de/iek_9_spectroscopy/ptma-ml).

## Supporting Information Available

Description of cross-validation, cross-validated error metrics for various regressors, optimized hyperparameters, description of error metrics, feature importance scores of the trained ERT-MBTR model, ESP fits, and details of *in operando* EPR experiments (PDF)
