# Supplementary material for: Machine Learning Isotropic g Values of Radical Polymers
Source: J Chem Theory Comput. 2024 Mar 8;20(6):2592–604. doi: 10.1021/acs.jctc.3c01252 (PMC10976631; doi:10.1021/acs.jctc.3c01252)
Supplement: Supplementary file 1 — ct3c01252_si_001.pdf [file ct3c01252_si_001.pdf]

Supporting Information

Machine learning isotropic g values of radical polymers

Davis Thomas Daniel,[*,†,‡] Souvik Mitra,[¶] Rüdiger-A. Eichel,[†,§] Diddo Diddens,[‖] and Josef Granwehr[†,‡]

†*Institute of Energy and Climate Research (IEK-9), Forschungszentrum Jülich GmbH, Jülich, 52425, Germany*

‡*Institute of Technical and Macromolecular Chemistry, RWTH Aachen University, Aachen, 52056, Germany*

¶*Institute of Physical Chemistry, University of Münster, Münster, 48149, Germany*

§*Institute of Physical Chemistry, RWTH Aachen University, Aachen 52056, Germany*

‖*Helmholtz-Institute Münster (IEK-12), Forschungszentrum Jülich GmbH, Jülich, Münster, 48149, Germany, 52425, Germany*

E-mail: d.daniel@fz-juelich.de

A1. Cross–validation

In five-fold cross-validation[1] (Figure S1), the **TR** data set is divided into 5 folds, out of which only 4 (circles with red outline) are used for training. The fold which is held out from training (circles with green outline labelled as 'Test') is then used as a test data set

for evaluation of the model. The process iterates 5 times and the final error metric is the average from evaluation of each of the 5 folds as test data set.

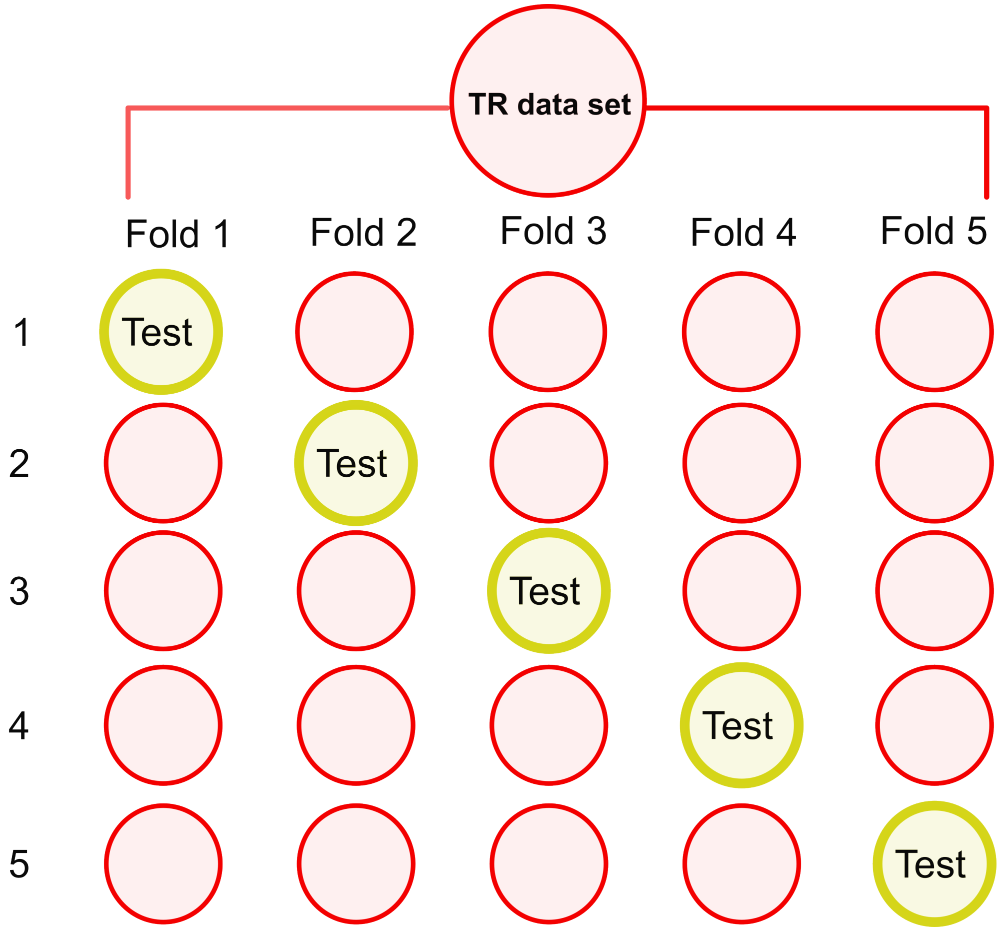

Figure S1: Pictorial representation of 5-fold cross validation. The **TR** data set is divided into 5 folds. At each iteration, 4 of the folds represented by circles with red outline are used for training the model and test fold denoted by circles with green outline are used for testing the model.

A2. Model selection

Figure S2 shows the different regressors evaluated for model selection. All models were used as implemented in *scikit-learn*.[2]

Hyperparameter optimization for all models was done using Grid search (GridsearchCV in *scikit-learn*) with 5-fold cross validation. For optimization, root mean squared error

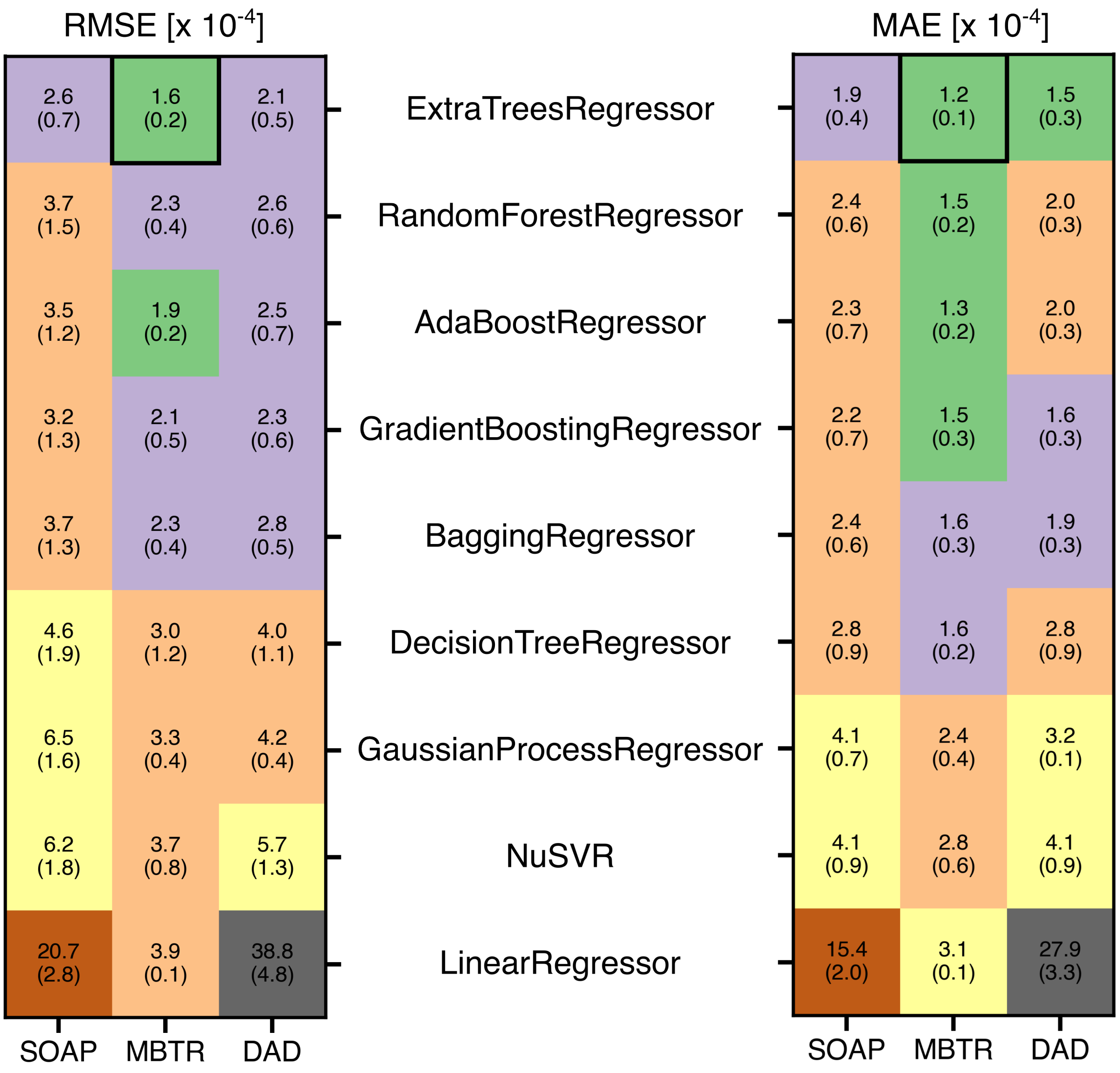

Figure S2: Cross validated error metrics obtained for different regressors using the **TR** dataset. RMSE values and standard deviation (inside parentheses) in units of 10^{-4} are shown on the left and MAE values and standard deviation (inside parentheses) in units of 10^{-4} are shown on the right.

(RMSE) was minimized. In case of Gaussian process regression, radial basis function (RBF) and rational quadratic kernels were optimized, and in case of support vector regression, linear kernel, RBF and polynomial kernels were optimized. For tree-based methods, hyper-parameters related to the number of trees and maximum depth of trees were optimized. For adaptive boosting regression and gradient boosting regression, learning rate was also opti-

mized. The error metrics (Figure S2) were computed by evaluating on the **TR** data set with 5–fold cross validated scoring (RMSE and MAE). For all three molecular representations, ExtraTreesRegressor (ERT) showed the best performance.

A3. Regression trees

ML models learn to correlate input variables, called features, with the desired output variables, such that the model can predict the output variable from unknown input variables. Generally, the input variables are represented by an array of features, called the feature vector. The data on which the model is trained, called training data set, consists of feature vectors and corresponding output variables. In the present context, feature vectors are derived from the molecular structure of PTMA and represented using SOAP, MBTR or DAD, while g_{iso} is the output variable.

Regression trees are built by recursively partitioning the feature space. For this purpose, a feature is selected and the feature space is split into nodes on the basis of a threshold that is compared with the value of the feature.[3,4] The split is decided in a way that within each node, the value of the output variable corresponding to the selected feature has the lowest variance. A commonly used criterion to decide the optimal split is the mean squared error (MSE) of the output variable within the node under consideration. The partitioning starts from the root node, containing m number of feature vectors of size n with corresponding values of the output variables, $G_{i\in[1,m]}$. For each feature $f_{i\in[1,n]}$ and a threshold value t, splits of the root node X into child nodes x_1 and x_2 are examined by evaluating the MSE of G within each child node x given by

$$\mathrm{MSE}_{x\in[x_1,x_2]} = \frac{1}{n_x} \sum_{G\in D(x)} \left(G - \bar{G}_x\right)^2 ,$$

where $D(x)$ denotes the subset of the training data in x and $\bar{G}_x$ is the mean of output variables corresponding to n_x number of feature vectors in x. The $f_{i\in[1,n]}$ and t value that

minimises the loss function, $L = \text{MSE}_{x_1} + \text{MSE}_{x_2}$, is used for splitting the node. The process of splitting is repeated at each of the successive child nodes until a stopping criterion is met.

Individual regression trees may lead to overfitting,[5] therefore an ensemble of randomised trees are used for more generalizability.[6] To obtain the final prediction, ensemble methods commonly utilise averaging[6,7] or boosting[8–10] techniques. In averaging techniques such as random forests, multiple models are trained independently on the same data set and the predictions from each model are averaged. In boosting techniques such as adaptive boosting, models are built sequentially. At each iteration of training, the samples in the training data set are assigned weights according to error of the current model in the sequence. The next models in the sequence are then trained on the weighted data set.

A common tree-based ensemble method, random forest[6] (RF), fits a number of regression trees, collectively called a forest, to fit the whole data set or a subset thereof. The final prediction is the arithmetic mean of the predictions from each tree.[11] In RF, features are randomly selected and an optimal threshold is found for each feature. The best split is then chosen according to the aforementioned splitting criterion. Another ensemble method, called extremely randomized trees[11] (ERT) introduces more randomness into the ensemble of trees. ERT differs from other tree-based algorithms, such as RF,[6] in the way the splits are decided. In ERT, both the features and the threshold are randomly selected, thereby reducing the computational cost of building the tree. Among the randomly generated splits, the best split is then chosen based on the splitting criterion. The tree is grown until a specified depth (number of nodes), or until each node contains the minimum number of allowed samples. For predictions, the decision path from the root node to the terminal nodes is followed, and the predicted value from each individual tree in the ensemble is averaged.

Tree-based models allow for the interpretation of how features influence the predictions obtained by a particular model through the calculation of feature importance scores. In ERT, importance scores are measured by the reduction in MSE achieved by splitting the tree on a particular feature. Such calculations may be more biased towards features with

more unique values and assign them higher importance scores.[12] Other approaches include calculation using permutation of features, wherein each feature is randomly shuffled and the change in error of the trained model is used as an importance measure.[6] However, if features are correlated, permuting a feature also affects the importance measure of features it is correlated to. This may result in misleading feature importance scores.[13]

A4. Hyperparameter optimisation

The hyperparameters optimised using grid search and 5-fold cross validation using the **TR** dataset are summarized in Table S1. For optimisation, root mean squared error (RMSE) was minimized.

Table S1: Hyperparameter optmisation and grid search bounds for the trained model using the **TR** dataset and 5-fold cross validation.

Parameter	Bounds	Optimal values
n_ estimators	range(10,110)	100
max_features	[None, sqrt, log2]	None
min_ samples_ split	[2, 4, 6]	2
min_ samples_ leaf	[2, 3, 4]	2
max_ depth	[None,range(10,40)]	10
max_ leaf_ nodes	[None,range(2,21)]	None
bootstrap	[True,False]	False

Another hyperparameter optimization strategy, using Optuna,[14] was also tested. Optimized hyperparameters using Optuna showed a decrease in the number trees (*n_ estimators*) and an increase in the depth of each tree (*max_ depth*) in comparison to hyperparameters optimized using grid search. Other parameters showed values similar to the hyperparameters optimized using grid search. Optimized hyperparameters from Optuna and the grid search are compared in Table S2. Performance metrics of the ERT models corresponding to the two optimization strategies are compared in Table S3. The final model was trained using the hyperparameters obtained from the grid search based on the better performance metrics.

Table S2: Optimized hyperparameters from Optuna and Grid search for the ERT-MBTR model.

Parameter	Optuna	Grid search
n_ estimators	58	100
max_features	None	None
min_ samples_ split	4	2
min_ samples_ leaf	3	2
max_ depth	38	10
max_ leaf_ nodes	None	None
bootstrap	False	False

Table S3: Five-fold cross validated error metrics for ERT-MBTR using the **TR** data set for different hyper parameter optimization strategies. Values inside parentheses denote the standard deviation.

Optimizer	R^2	MAE	RMSE
Optuna	0.94 (0.02)	1.30 (0.21)	1.76 (0.34)
Grid search	0.95 (0.02)	1.25 (0.14)	1.58 (0.20)

A5. Error Metrics

The coefficient of determination (R^2) is given by,

$$R^2 = 1 - \sum_{i=1}^{n} \left(g_i^{\text{calc}} - g_i^{\text{pred}} \right)^2 / \sum_{i=1}^{n} \left(g_i^{\text{calc}} - \bar{g}^{\text{calc}} \right)^2 \tag{1}$$

where DFT-calculated g values are denoted by g^{calc}, predicted g values as g^{pred}, and n is the number of samples. R^2 is a measure of how well a particular data set is fit by the model. Values range between 0 and 1, and $R^2 = 1$ implies an optimal fit. As R^2 is dependent on the variance in the data set, the metric may not be valid for comparing model performance on different data sets, therefore MAE and RMSE are also provided.

MAE and RMSE metrics describe the error of residuals and are given by

$$\text{MAE} = \frac{1}{n} \sum_{i=1}^{n} \left(|g_i^{\text{calc}} - g_i^{\text{pred}}| \right) , \tag{2}$$

$$\text{RMSE} = \sqrt{\frac{1}{n}\sum_{i=1}^{n}\left(g_i^{\text{calc}} - g_i^{\text{pred}}\right)^2}. \tag{3}$$

Both MAE and RMSE have a similar scale to the input data set but RMSE penalises outliers more heavily than MAE. Since the dynamic range of the data set used in this study is small, a calculation of both RMSE and MAE is recommended.[15,16]

A6. Molecular descriptors

For the ERT model, dependence of model performance on the cutoff distance (r_{cut}) for SOAP and MBTR was studied using 5-fold cross validation with the **TR** dataset. Table S4 summarizes the cross-validated error metrics. The cutoff distance used in the construction of the final model was chosen based on RMSE.

Table S4: Dependence of model performance on r_{cut}. Optimization was using the **TR** dataset and 5-fold cross validation.Values inside parentheses denote the standard deviation.

Molecular descriptor	Parameter	R^2	MAE	RMSE
SOAP_1	$r_{\text{cut}} = 5$	0.81 (0.13)	2.07 (0.35)	3.06 (0.80)
SOAP_2	$r_{\text{cut}} = 10$	0.87 (0.09)	1.91 (0.37)	2.59 (0.68)
SOAP_3	$r_{\text{cut}} = 20$	0.83 (0.09)	2.04 (0.26)	3.06 (0.72)
MBTR_1	$r_{\text{cut}}(k_2) = 5$	0.95 (0.02)	1.25 (0.14)	1.60 (0.20)
MBTR_2	$r_{\text{cut}}(k_2) = 10$	0.95 (0.02)	1.25 (0.14)	1.58 (0.20)
MBTR_3	$r_{\text{cut}}(k_2) = 20$	0.95 (0.02)	1.24 (0.14)	1.58 (0.17)

Dependence of the ERT model performance on the feature vector size of SOAP and MBTR representations are summarized in Table S5. For MBTR, the vector size was changed by changing the number of discretization points of grids that define different features in a systematic manner. For SOAP, the size was changed by changing the number of radial basis functions and degree of spherical harmonics. For different feature vector sizes of MBTR and SOAP, there was no significant change in model performance with the size of the feature vector for a particular descriptor. Additionally, for comparable feature vector sizes, better performance exhibited by the ERT-MBTR model is maintained. In case of SOAP, with

larger vector sizes, the model exhibits a slight drop in performance which is likely due to over-fitting to the training data.

Table S5: Dependence of ERT model performance on SOAP and MBTR feature vector sizes. Optimization was using the **TR** dataset and 5-fold cross validation. Values inside parentheses denote the standard deviation.

Molecular descriptor	Size	R^2	MAE	RMSE
SOAP	7992	0.85 (0.12)	1.93 (0.35)	2.71 (0.71)
SOAP	4752	0.87 (0.09)	1.91 (0.37)	2.59 (0.68)
SOAP	2100	0.85 (0.09)	2.09 (0.11)	2.82 (0.26)
MBTR	7740	0.95 (0.02)	1.25 (0.14)	1.58 (0.20)
MBTR	4810	0.95 (0.02)	1.23 (0.15)	1.62 (0.22)
MBTR	2090	0.94 (0.03)	1.27 (0.17)	1.66 (0.25)

A7. Feature importance scores

Figure S3 show the feature importance scores for the ERT-DAD model. For simplicity, feature importances belonging to the same feature group (e.g. all indexes which belong to N−O) are summed.

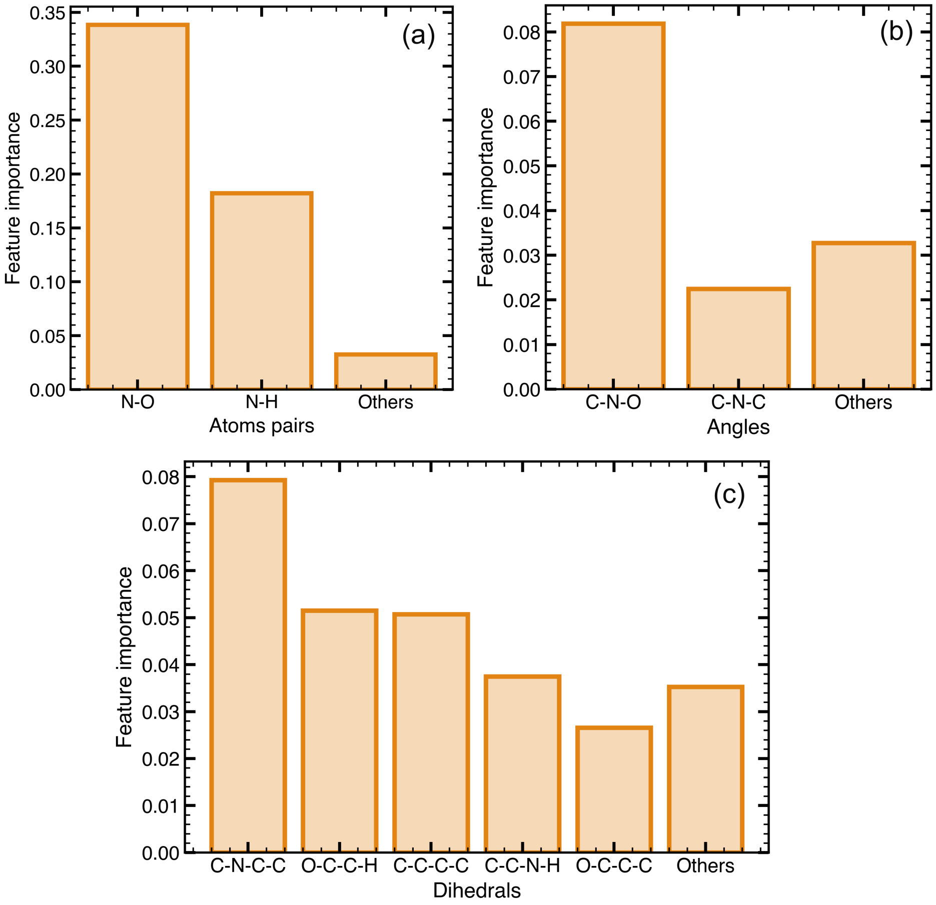

Figure S3: Feature importance scores for DAD for (a) distance features, (b) angular features, and (c) dihedral features

A8. Comparison of MBTR outputs

The MBTR outputs of PTMA–4 structures from the **TE-2** data set corresponding to time frames of 0 ps, 1.0x10^4 ps, 1.5x10^4 ps and 2.0x10^4 ps are compared in Figure S4. The region of MBTR outputs corresponding to important features of N−O distances and H−C−C angles show minimal differences in cases of time frames after $g_{\mathrm{iso}}^{\mathrm{calc}}$ converges. In comparison, unrelaxed structure at t=0 shows considerable difference.

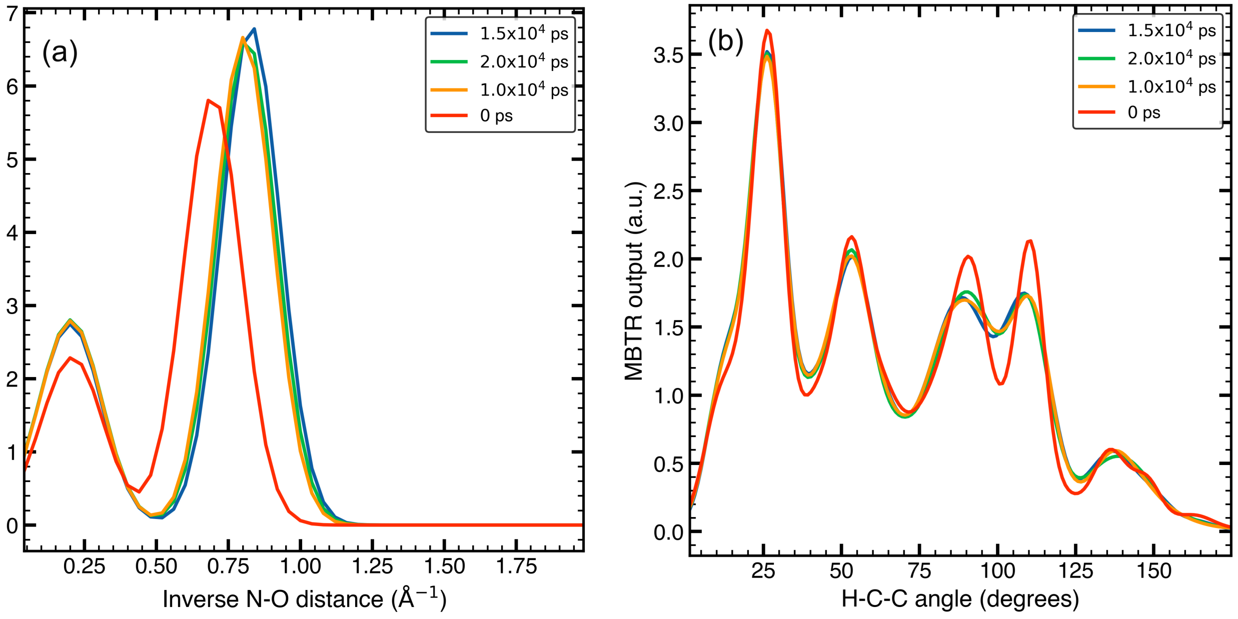

Figure S4: MBTR outputs of two PTMA-6 structures from the **TE-2** data set corresponding to time frames of of 0 ps, 1.0x10^4 ps, 1.5x10^4 ps and 2.0x10^4 ps. (a) N−O distance distribution of the MBTR outputs. (b) H−C−C angular distribution of the MBTR output.

A9. Molecular Dynamics

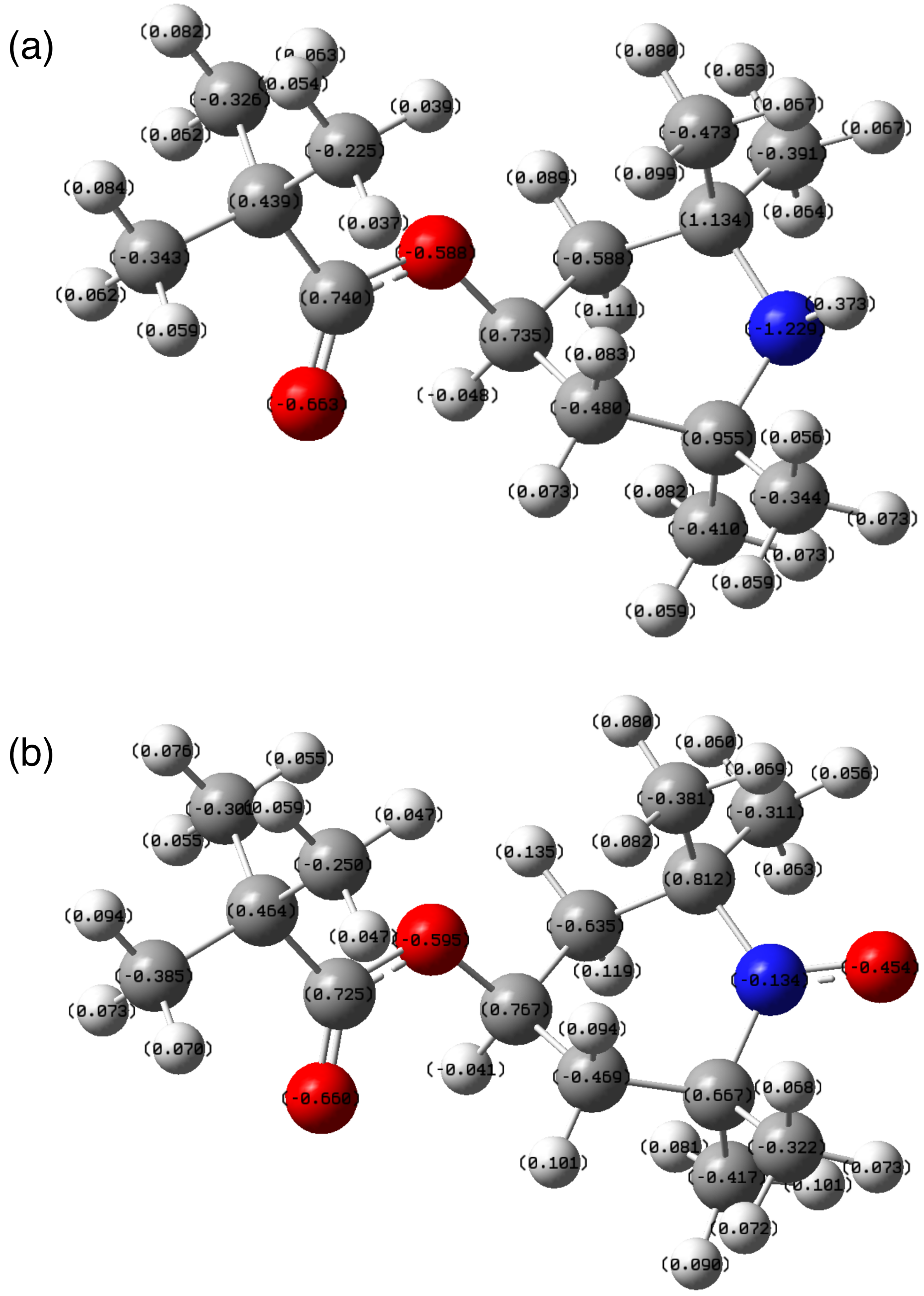

Figure S5: Atomic site charges of (a) diamagnetic and (b) paramagnetic repeat units of PTMA from the electrostatic potential (ESP) fit.

A10. *In operando* EPR spectroscopy of a PTMA-based ORB

Materials and methods

The *in operando* cell design by Niemöller *et. al.* was employed.[17] As anode, a circular Li foil of 380 µm thickness and 7 mm diameter was used. PTMA-based cathodes were punched out as 7 mm diameter discs from a 200 µm thick PTMA composite (60 wt.% of PTMA, 35 wt.% Super P and 5 wt.% of carboxymethyl cellulose binder) coated on a 20 µm thick Al foil. The electrode discs were stacked together with a 25 µm Celgard 2500 separator of 8 mm diameter in between. 25 µL LP57 (1M $LiPF_6$ in EC (ethylene carbonate):EMC (ethyl methyl carbonate) 3:7) was used as electrolyte. The cell housing also included a ruby as reference for phase and amplitude correction. The assembled cell was transferred to a 8 mm outer diameter EPR tube. The entire cell preparation was done inside an argon-filled glove box.

Battery cycling tests were done with a Biologic SP-200 potentiostat. Charging and discharging were performed between 3.0 V and 4.2 V in constant current mode at 0.4C (20 µA).

X-Band CW EPR spectra were recorded using a Bruker Elexsys E540 EPR spectrometer operating at 9.26 GHz equipped with a 4108 TMHS resonator. The spectra were recorded at room temperature as first derivatives of the absorption function, with a field modulation amplitude of 0.15 mT, modulation frequency of 100 kHz, and microwave power of 2.0 mW. Phase, amplitude and the resonance field shift due to impedance changes in the microwave cavity was corrected using the ruby reference signal. To extract $g_{\mathrm{iso}}^{\mathrm{exp}}$ and EPR integral intensity of PTMA, the EPR signal was fit using the sum of two phase-adaptable Lorentzian functions corresponding to contributions from PTMA and carbon black.

Variation of $g_{\text{iso}}^{\text{exp}}$ with state of charge

Figure S6a and S6b shows the variation of $g_{\text{iso}}^{\text{exp}}$ and EPR integral intensity, respectively, of PTMA as a function of cell potential. In the pristine state and in the state of highest radical density at the reduction limit (3.0 V), $g_{\text{iso}}^{\text{exp}}$ of PTMA is 2.0065. Near the oxidation limit (4.2 V), the contribution from the carbon black component dominates the EPR spectrum and the PTMA component is broad and overlaps with the carbon black component. Therefore, the obtained $g_{\text{iso}}^{\text{exp}}$ shows more noise as the oxidation limit is approached. A manual inspection of EPR spectra near the oxidation limit revealed that the $g_{\text{iso}}^{\text{exp}}$ is largely constant near the oxidation limit and is approximately equal to 2.0040.

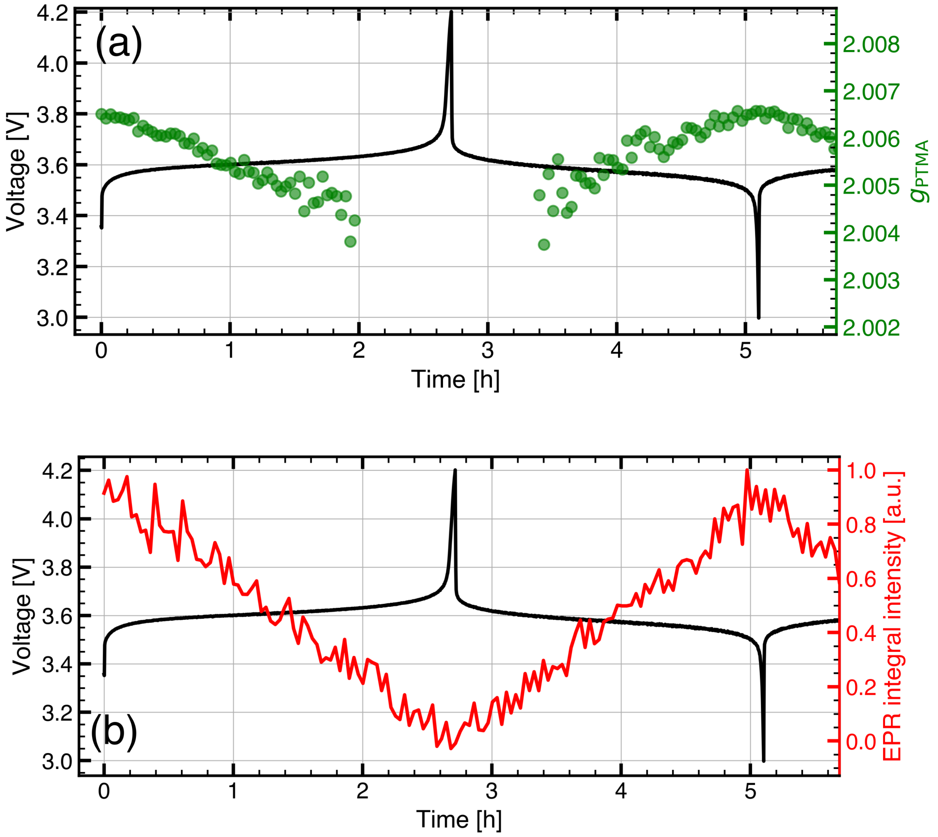

Figure S6: Variation of (a) $g_{\text{iso}}^{\text{exp}}$ and (b) the EPR integral intensity with the cell potential for a PTMA-based ORB cycled at 0.4C.

References

(1) Fushiki, T. Estimation of prediction error by using K-fold cross-validation. *Statistics and Computing* **2011**, *21*, 137–146.

(2) Pedregosa, F.; Varoquaux, G.; Gramfort, A.; Michel, V.; Thirion, B.; Grisel, O.; Blondel, M. et al. Scikit-learn: Machine Learning in Python. *Journal of Machine Learning Research* **2011**, *12*, 2825–2830.

(3) Breiman, L.; Friedman, J.; Stone, C. J.; Olshen, R. A. *Classification and regression trees*; CRC press, 1984.

(4) Loh, W.-Y. Classification and regression trees. *Wiley Interdisciplinary Reviews: Data Mining and Knowledge Discovery* **2011**, *1*, 14–23.

(5) Geurts, P.; Irrthum, A.; Wehenkel, L. Supervised learning with decision tree-based methods in computational and systems biology. *Molecular Biosystems* **2009**, *5*, 1593–1605.

(6) Breiman, L. Random forests. *Machine Learning* **2001**, *45*, 5–32.

(7) Breiman, L. Bagging predictors. *Machine Learning* **1996**, *24*, 123–140.

(8) Freund, Y.; Schapire, R. E. A decision-theoretic generalization of on-line learning and an application to boosting. *Journal of Computer and System Sciences* **1997**, *55*, 119–139.

(9) Friedman, J. H. Stochastic gradient boosting. *Computational Statistics & Data Analysis* **2002**, *38*, 367–378.

(10) Drucker, H. Improving regressors using boosting techniques. Icml. 1997; pp 107–115.

(11) Geurts, P.; Ernst, D.; Wehenkel, L. Extremely randomized trees. *Machine Learning* **2006**, *63*, 3–42.

(12) Strobl, C.; Boulesteix, A.-L.; Zeileis, A.; Hothorn, T. Bias in random forest variable importance measures: Illustrations, sources and a solution. *BMC Bioinformatics* **2007**, *8*, 1–21.

(13) Hooker, G.; Mentch, L.; Zhou, S. Unrestricted permutation forces extrapolation: variable importance requires at least one more model, or there is no free variable importance. *Statistics and Computing* **2021**, *31*, 1–16.

(14) Akiba, T.; Sano, S.; Yanase, T.; Ohta, T.; Koyama, M. Optuna: A Next-generation Hyperparameter Optimization Framework. Proceedings of the 25th ACM SIGKDD International Conference on Knowledge Discovery and Data Mining. 2019.

(15) Bender, A.; Schneider, N.; Segler, M.; Patrick Walters, W.; Engkvist, O.; Rodrigues, T. Evaluation guidelines for machine learning tools in the chemical sciences. *Nature Reviews Chemistry* **2022**, *6*, 428–442.

(16) Pernot, P.; Huang, B.; Savin, A. Impact of non-normal error distributions on the benchmarking and ranking of Quantum Machine Learning models. *Machine Learning: Science and Technology* **2020**, *1*, 035011.

(17) Niemöller, A.; Jakes, P.; Eurich, S.; Paulus, A.; Kungl, H.; Eichel, R.-A.; Granwehr, J. Monitoring local redox processes in $LiNi_{0.5}Mn_{1.5}O_4$ battery cathode material by in operando EPR spectroscopy. *The Journal of Chemical Physics* **2018**, *148*.
